# Supplementary material for: The Impact of the Localization of Metastasis in Bladder Cancer Patients with Recurrence After Cystectomy
Source: Cancers (Basel). 2025 Mar 3;17(5):867. doi: 10.3390/cancers17050867 (PMC11899092; doi:10.3390/cancers17050867)
Supplement: Supplementary file 1 [file cancers-17-00867-s001.zip › cancers-3460651-supplementary.pdf]

Table S1. All Cystectomies 2015-2021

Characteristic	No Recurrence N = 484[1]	Recurrence N = 180[1]	p-value[2]
**Sex**			0.2
Male	358 (74%)	124 (69%)	
Female	126 (26%)	56 (31%)	
**Age**	72 (66, 77)	73 (66, 78)	0.2
**BMI**			0.3
Unknown	157 (32%)	65 (36%)	
<25	217 (45%)	79 (44%)	
25-29.9	102 (21%)	36 (20%)	
>30	8 (1.7%)	<4 (<2.2%)	
**ASA classification**			0.3
Unknown	9 (1.9%)	<4 (<2.2%)	
1	39 (8.1%)	13 (7.2%)	
2	290 (60%)	105 (58%)	
3	144 (30%)	62 (34%)	
4	<4 (<0.4%)	<4 (<2.2%)	
**Smoking status**			0.5
Never	73 (15%)	33 (18%)	
>5years ago	197 (41%)	75 (42%)	
<5years ago	55 (11%)	16 (8.9%)	
Smoker	135 (28%)	51 (28%)	
Unknown	24 (5.0%)	5 (2.8%)	
**Neoadjuvant Chemotherapy**			0.005
Neoadjuvant chemo	126 (26%)	67 (37%)	
No Neoadjuvant chemo	358 (74%)	113 (63%)	
**BCG treatment**			0.8
BCG	46 (9.5%)	16 (8.9%)	
No BCG	438 (90%)	164 (91%)	
**Robot assisted**	242 (50%)	80 (44%)	0.2
**Urinary Conduit**			>0.9

Characteristic	No Recurrence N = 484[1]	Recurrence N = 180[1]	p-value[2]
Ileal conduit ad Modum Bricker	458 (95%)	170 (94%)	
Other	26 (5.4%)	10 (5.6%)	
**Tumor stage (TURB)**			<0.001
Unknown	18 (3.7%)	<4 (<2.2%)	
Ta, CIS or T1	198 (41%)	44 (24%)	
T2+	268 (55%)	135 (75%)	
**Tumor stage (Cystectomy)**			
Unknown	15 (3.1%)	<4 (<2.2%)	
No tumor left	207 (43%)	29 (16%)	
Ta	22 (4.5%)	4 (2.2%)	
CIS	79 (16%)	4 (2.2%)	
T1	41 (8.5%)	9 (5.0%)	
T2	49 (10%)	16 (8.9%)	
≥T3	71 (15%)	116 (64%)	
**Lymph Nodes Removed**	25 (17, 33)	24 (15, 34)	0.4
**Nodal Status**			<0.001
Positive	33 (6.8%)	60 (33%)	
Negative	451 (93%)	120 (67%)	

[1] n (%); Median (IQR)

[2] Pearson’s Chi-squared test; Wilcoxon rank sum test; Fisher’s exact test

Table S2. Single vs. Multiple Recurrence

Characteristic	**Single Recurrence**, N = 72[1]	**≥2 Recurrences**, N = 108[1]	**p-value**[2]
**Sex**			0.15
Male	54 (75%)	70 (65%)	
Female	18 (25%)	38 (35%)	
**Age**	75 (68, 78)	72 (66, 77)	0.3
**BMI**			0.6
<25	28 (39%)	37 (34%)	
25-29.9	32 (44%)	47 (44%)	
>30	12 (17%)	24 (22%)	
**ASA classification**			0.6
1	5 (6.9%)	8 (7.4%)	
2	39 (54%)	66 (61%)	
3	28 (39%)	34 (31%)	
**Smoking status**			0.5
Never	14 (19%)	19 (18%)	
>5years ago	29 (40%)	46 (43%)	
<5years ago	4 (5.6%)	12 (11%)	
Smoker	24 (33%)	27 (25%)	
Unknown	<4 (<5.5%)	4 (3.7%)	
**Neoadjuvant Chemotherapy**			0.4
Neoadjuvant chemo	24 (33%)	43 (40%)	
No Neoadjuvant chemo	48 (67%)	65 (60%)	
**BCG treatment**			0.7
BCG	7 (9.7%)	9 (8.3%)	
No BCG	65 (90%)	99 (92%)	
**Robot assisted**	39 (54%)	41 (38%)	0.032

**Characteristic**	**Single Recurrence**, N = 72[1]	**≥2 Recurrences**, N = 108[1]	**p-value**[2]
**Urinary Conduit**			0.7
Ileal conduit ad Modum Bricker	69 (96%)	101 (94%)	
Other	<4 (<5.5%)	7 (6.5%)	
**Tumor stage (TURB)**			0.4
Unknown	<4 (<5.5%)	<4 (<3.7%)	
Ta, CIS or T1	19 (26%)	25 (23%)	
T2+	52 (72%)	83 (77%)	
**Tumor stage (Cystectomy)**			0.2
Unkown	<4 (<5.5%)	<4 (<3.7%)	
No tumor left	14 (19%)	15 (14%)	
Ta	<4 (<5.5%)	<4 (<3.7%)	
CIS	<4 (<5.5%)	<4 (<3.7%)	
T1	<4 (<5.5%)	8 (7.4%)	
T2	8 (11%)	8 (7.4%)	
≥T3	43 (60%)	73 (68%)	
**Lymph Nodes Removed**	25 (15, 36)	24 (14, 33)	0.5
**Nodal Status**			>0.9
Positive	24 (33%)	36 (33%)	
Negative	48 (67%)	72 (67%)	

[1] n (%); Median (IQR)

[2] Pearson's Chi-squared test; Wilcoxon rank sum test; Fisher's exact test
